# Evaluating different methods of microarray data normalization

**DOI:** 10.1186/1471-2105-7-469

**Published:** 2006-10-23

**Authors:** André Fujita, João Ricardo Sato, Leonardo de Oliveira Rodrigues, Carlos Eduardo Ferreira, Mari Cleide Sogayar

**Affiliations:** 1 Institute of Mathematics and Statistics, University of São Paulo, Rua do Matão, 1010 – São Paulo, 05508-090 SP, Brazil; 2 Chemistry Institute, University of São Paulo, Av. Lineu Prestes, 748 – São Paulo, 05513-970 SP, Brazil

## Abstract

**Background:**

With the development of DNA hybridization microarray technologies, nowadays it is possible to simultaneously assess the expression levels of thousands to tens of thousands of genes. Quantitative comparison of microarrays uncovers distinct patterns of gene expression, which define different cellular phenotypes or cellular responses to drugs. Due to technical biases, normalization of the intensity levels is a pre-requisite to performing further statistical analyses. Therefore, choosing a suitable approach for normalization can be critical, deserving judicious consideration.

**Results:**

Here, we considered three commonly used normalization approaches, namely: Loess, Splines and Wavelets, and two non-parametric regression methods, which have yet to be used for normalization, namely, the Kernel smoothing and Support Vector Regression. The results obtained were compared using artificial microarray data and benchmark studies. The results indicate that the Support Vector Regression is the most robust to outliers and that Kernel is the worst normalization technique, while no practical differences were observed between Loess, Splines and Wavelets.

**Conclusion:**

In face of our results, the Support Vector Regression is favored for microarray normalization due to its superiority when compared to the other methods for its robustness in estimating the normalization curve.

## Background

DNA microarray technology is a powerful approach for genomic research, playing an increasingly important role in biomedical research. This technology yields simultaneous measurement of gene expression levels of thousands of genes, allowing the analysis of differential gene expression patterns under different conditions such as disease (pathological) states or treatment with different chemotherapeutic drugs. Due to small differences in RNA quantities and fluctuations generated by the technique, the intensity levels may vary from one replicate to the other due to effects which are unrelated to the genes, requiring data normalization before they can be compared.

Therefore, normalization is an important step for microarray data analysis. The purpose of data normalization is to minimize the effects caused by technical variations and, as a result, allow the data to be comparable in order to find actual biological changes. Several normalization approaches have been proposed, most of which derive from studies using two-color spotted microarrays. Some authors proposed normalization of the hybridization intensity ratios; others use global, linear methods, while others use local, non-linear methods. Several authors suggested using the spike-in controls, housekeeping genes, or invariant genes [1-7].

Recently, some authors suggested the use of non-linear normalization methods [8-10] which are believed to be superior to the above mentioned approaches. The locally weighed regression Lowess procedure [11] has been widely used for this purpose and implemented by several microarray analysis software packages [12,13], but similar methods are suggested such as Splines [14,15] and Wavelets [16].

Here, we compare three different well-known microarray data normalization methods, namely: Loess Regression (LR), Splines Smoothing (SS) and Wavelets Smoothing (WS). In addition, we propose two different normalization approaches, called Kernel Regression (KR) [17,18] and Support Vector Regression (SVR) [19], which, to the best of our knowledge, have yet to be applied for microarray normalization. In order to assess the most appropriate normalization technique, benchmark studies were carried out using data derived from CodeLink™ mouse microarray experiments [20], generated at our Cell and Molecular Biology Laboratory (Chemistry Institute, University of São Paulo).

## Results

We sought to highlight the performance of five different methods of microarray normalization, namely: Loess, Splines, Wavelets, Kernel and Support Vector Regression in a simulated microarray and in an actual CodeLink™ microarray platform, which comprised ten thousand mouse genes. Although we have focused on the use of simulated two-color cDNA microarray data analysis, our discussions are also applicable to the single-color oligonucleotide microarrays.

The artificial microarrays composed by ten thousand spots were generated using the model proposed by Balagurunathan et al. (2002) [21]. The parameters used were: (a01
 MathType@MTEF@5@5@+=feaafiart1ev1aaatCvAUfKttLearuWrP9MDH5MBPbIqV92AaeXatLxBI9gBaebbnrfifHhDYfgasaacH8akY=wiFfYdH8Gipec8Eeeu0xXdbba9frFj0=OqFfea0dXdd9vqai=hGuQ8kuc9pgc9s8qqaq=dirpe0xb9q8qiLsFr0=vr0=vr0dc8meaabaqaciaacaGaaeqabaqabeGadaaakeaacqWGHbqydaqhaaWcbaGaeGimaadabaGaeGymaedaaaaa@3002@ = 0, a11
 MathType@MTEF@5@5@+=feaafiart1ev1aaatCvAUfKttLearuWrP9MDH5MBPbIqV92AaeXatLxBI9gBaebbnrfifHhDYfgasaacH8akY=wiFfYdH8Gipec8Eeeu0xXdbba9frFj0=OqFfea0dXdd9vqai=hGuQ8kuc9pgc9s8qqaq=dirpe0xb9q8qiLsFr0=vr0=vr0dc8meaabaqaciaacaGaaeqabaqabeGadaaakeaacqWGHbqydaqhaaWcbaGaeGymaedabaGaeGymaedaaaaa@3004@ = 100^1/0.7^, a21
 MathType@MTEF@5@5@+=feaafiart1ev1aaatCvAUfKttLearuWrP9MDH5MBPbIqV92AaeXatLxBI9gBaebbnrfifHhDYfgasaacH8akY=wiFfYdH8Gipec8Eeeu0xXdbba9frFj0=OqFfea0dXdd9vqai=hGuQ8kuc9pgc9s8qqaq=dirpe0xb9q8qiLsFr0=vr0=vr0dc8meaabaqaciaacaGaaeqabaqabeGadaaakeaacqWGHbqydaqhaaWcbaGaeGOmaidabaGaeGymaedaaaaa@3006@ = -0.7, a31
 MathType@MTEF@5@5@+=feaafiart1ev1aaatCvAUfKttLearuWrP9MDH5MBPbIqV92AaeXatLxBI9gBaebbnrfifHhDYfgasaacH8akY=wiFfYdH8Gipec8Eeeu0xXdbba9frFj0=OqFfea0dXdd9vqai=hGuQ8kuc9pgc9s8qqaq=dirpe0xb9q8qiLsFr0=vr0=vr0dc8meaabaqaciaacaGaaeqabaqabeGadaaakeaacqWGHbqydaqhaaWcbaGaeG4mamdabaGaeGymaedaaaaa@3008@ = 1) and (a02
 MathType@MTEF@5@5@+=feaafiart1ev1aaatCvAUfKttLearuWrP9MDH5MBPbIqV92AaeXatLxBI9gBaebbnrfifHhDYfgasaacH8akY=wiFfYdH8Gipec8Eeeu0xXdbba9frFj0=OqFfea0dXdd9vqai=hGuQ8kuc9pgc9s8qqaq=dirpe0xb9q8qiLsFr0=vr0=vr0dc8meaabaqaciaacaGaaeqabaqabeGadaaakeaacqWGHbqydaqhaaWcbaGaeGimaadabaGaeGOmaidaaaaa@3004@ = 0, a12
 MathType@MTEF@5@5@+=feaafiart1ev1aaatCvAUfKttLearuWrP9MDH5MBPbIqV92AaeXatLxBI9gBaebbnrfifHhDYfgasaacH8akY=wiFfYdH8Gipec8Eeeu0xXdbba9frFj0=OqFfea0dXdd9vqai=hGuQ8kuc9pgc9s8qqaq=dirpe0xb9q8qiLsFr0=vr0=vr0dc8meaabaqaciaacaGaaeqabaqabeGadaaakeaacqWGHbqydaqhaaWcbaGaeGymaedabaGaeGOmaidaaaaa@3006@ = 100^1/0.9^, a22
 MathType@MTEF@5@5@+=feaafiart1ev1aaatCvAUfKttLearuWrP9MDH5MBPbIqV92AaeXatLxBI9gBaebbnrfifHhDYfgasaacH8akY=wiFfYdH8Gipec8Eeeu0xXdbba9frFj0=OqFfea0dXdd9vqai=hGuQ8kuc9pgc9s8qqaq=dirpe0xb9q8qiLsFr0=vr0=vr0dc8meaabaqaciaacaGaaeqabaqabeGadaaakeaacqWGHbqydaqhaaWcbaGaeGOmaidabaGaeGOmaidaaaaa@3008@ = -0.9, a32
 MathType@MTEF@5@5@+=feaafiart1ev1aaatCvAUfKttLearuWrP9MDH5MBPbIqV92AaeXatLxBI9gBaebbnrfifHhDYfgasaacH8akY=wiFfYdH8Gipec8Eeeu0xXdbba9frFj0=OqFfea0dXdd9vqai=hGuQ8kuc9pgc9s8qqaq=dirpe0xb9q8qiLsFr0=vr0=vr0dc8meaabaqaciaacaGaaeqabaqabeGadaaakeaacqWGHbqydaqhaaWcbaGaeG4mamdabaGaeGOmaidaaaaa@300A@ = 1) for sinusoid shape, (a01
 MathType@MTEF@5@5@+=feaafiart1ev1aaatCvAUfKttLearuWrP9MDH5MBPbIqV92AaeXatLxBI9gBaebbnrfifHhDYfgasaacH8akY=wiFfYdH8Gipec8Eeeu0xXdbba9frFj0=OqFfea0dXdd9vqai=hGuQ8kuc9pgc9s8qqaq=dirpe0xb9q8qiLsFr0=vr0=vr0dc8meaabaqaciaacaGaaeqabaqabeGadaaakeaacqWGHbqydaqhaaWcbaGaeGimaadabaGaeGymaedaaaaa@3002@ = 0, a11
 MathType@MTEF@5@5@+=feaafiart1ev1aaatCvAUfKttLearuWrP9MDH5MBPbIqV92AaeXatLxBI9gBaebbnrfifHhDYfgasaacH8akY=wiFfYdH8Gipec8Eeeu0xXdbba9frFj0=OqFfea0dXdd9vqai=hGuQ8kuc9pgc9s8qqaq=dirpe0xb9q8qiLsFr0=vr0=vr0dc8meaabaqaciaacaGaaeqabaqabeGadaaakeaacqWGHbqydaqhaaWcbaGaeGymaedabaGaeGymaedaaaaa@3004@ = 500 a21
 MathType@MTEF@5@5@+=feaafiart1ev1aaatCvAUfKttLearuWrP9MDH5MBPbIqV92AaeXatLxBI9gBaebbnrfifHhDYfgasaacH8akY=wiFfYdH8Gipec8Eeeu0xXdbba9frFj0=OqFfea0dXdd9vqai=hGuQ8kuc9pgc9s8qqaq=dirpe0xb9q8qiLsFr0=vr0=vr0dc8meaabaqaciaacaGaaeqabaqabeGadaaakeaacqWGHbqydaqhaaWcbaGaeGOmaidabaGaeGymaedaaaaa@3006@ = -1, a31
 MathType@MTEF@5@5@+=feaafiart1ev1aaatCvAUfKttLearuWrP9MDH5MBPbIqV92AaeXatLxBI9gBaebbnrfifHhDYfgasaacH8akY=wiFfYdH8Gipec8Eeeu0xXdbba9frFj0=OqFfea0dXdd9vqai=hGuQ8kuc9pgc9s8qqaq=dirpe0xb9q8qiLsFr0=vr0=vr0dc8meaabaqaciaacaGaaeqabaqabeGadaaakeaacqWGHbqydaqhaaWcbaGaeG4mamdabaGaeGymaedaaaaa@3008@ = 1) and (a02
 MathType@MTEF@5@5@+=feaafiart1ev1aaatCvAUfKttLearuWrP9MDH5MBPbIqV92AaeXatLxBI9gBaebbnrfifHhDYfgasaacH8akY=wiFfYdH8Gipec8Eeeu0xXdbba9frFj0=OqFfea0dXdd9vqai=hGuQ8kuc9pgc9s8qqaq=dirpe0xb9q8qiLsFr0=vr0=vr0dc8meaabaqaciaacaGaaeqabaqabeGadaaakeaacqWGHbqydaqhaaWcbaGaeGimaadabaGaeGOmaidaaaaa@3004@ = 0, a12
 MathType@MTEF@5@5@+=feaafiart1ev1aaatCvAUfKttLearuWrP9MDH5MBPbIqV92AaeXatLxBI9gBaebbnrfifHhDYfgasaacH8akY=wiFfYdH8Gipec8Eeeu0xXdbba9frFj0=OqFfea0dXdd9vqai=hGuQ8kuc9pgc9s8qqaq=dirpe0xb9q8qiLsFr0=vr0=vr0dc8meaabaqaciaacaGaaeqabaqabeGadaaakeaacqWGHbqydaqhaaWcbaGaeGymaedabaGaeGOmaidaaaaa@3006@ = 10, a22
 MathType@MTEF@5@5@+=feaafiart1ev1aaatCvAUfKttLearuWrP9MDH5MBPbIqV92AaeXatLxBI9gBaebbnrfifHhDYfgasaacH8akY=wiFfYdH8Gipec8Eeeu0xXdbba9frFj0=OqFfea0dXdd9vqai=hGuQ8kuc9pgc9s8qqaq=dirpe0xb9q8qiLsFr0=vr0=vr0dc8meaabaqaciaacaGaaeqabaqabeGadaaakeaacqWGHbqydaqhaaWcbaGaeGOmaidabaGaeGOmaidaaaaa@3008@ = -1, a32
 MathType@MTEF@5@5@+=feaafiart1ev1aaatCvAUfKttLearuWrP9MDH5MBPbIqV92AaeXatLxBI9gBaebbnrfifHhDYfgasaacH8akY=wiFfYdH8Gipec8Eeeu0xXdbba9frFj0=OqFfea0dXdd9vqai=hGuQ8kuc9pgc9s8qqaq=dirpe0xb9q8qiLsFr0=vr0=vr0dc8meaabaqaciaacaGaaeqabaqabeGadaaakeaacqWGHbqydaqhaaWcbaGaeG4mamdabaGaeGOmaidaaaaa@300A@ = 1) for banana shape and, (a01
 MathType@MTEF@5@5@+=feaafiart1ev1aaatCvAUfKttLearuWrP9MDH5MBPbIqV92AaeXatLxBI9gBaebbnrfifHhDYfgasaacH8akY=wiFfYdH8Gipec8Eeeu0xXdbba9frFj0=OqFfea0dXdd9vqai=hGuQ8kuc9pgc9s8qqaq=dirpe0xb9q8qiLsFr0=vr0=vr0dc8meaabaqaciaacaGaaeqabaqabeGadaaakeaacqWGHbqydaqhaaWcbaGaeGimaadabaGaeGymaedaaaaa@3002@ = 0, a11
 MathType@MTEF@5@5@+=feaafiart1ev1aaatCvAUfKttLearuWrP9MDH5MBPbIqV92AaeXatLxBI9gBaebbnrfifHhDYfgasaacH8akY=wiFfYdH8Gipec8Eeeu0xXdbba9frFj0=OqFfea0dXdd9vqai=hGuQ8kuc9pgc9s8qqaq=dirpe0xb9q8qiLsFr0=vr0=vr0dc8meaabaqaciaacaGaaeqabaqabeGadaaakeaacqWGHbqydaqhaaWcbaGaeGymaedabaGaeGymaedaaaaa@3004@ = 10, a21
 MathType@MTEF@5@5@+=feaafiart1ev1aaatCvAUfKttLearuWrP9MDH5MBPbIqV92AaeXatLxBI9gBaebbnrfifHhDYfgasaacH8akY=wiFfYdH8Gipec8Eeeu0xXdbba9frFj0=OqFfea0dXdd9vqai=hGuQ8kuc9pgc9s8qqaq=dirpe0xb9q8qiLsFr0=vr0=vr0dc8meaabaqaciaacaGaaeqabaqabeGadaaakeaacqWGHbqydaqhaaWcbaGaeGOmaidabaGaeGymaedaaaaa@3006@ = -1, a31
 MathType@MTEF@5@5@+=feaafiart1ev1aaatCvAUfKttLearuWrP9MDH5MBPbIqV92AaeXatLxBI9gBaebbnrfifHhDYfgasaacH8akY=wiFfYdH8Gipec8Eeeu0xXdbba9frFj0=OqFfea0dXdd9vqai=hGuQ8kuc9pgc9s8qqaq=dirpe0xb9q8qiLsFr0=vr0=vr0dc8meaabaqaciaacaGaaeqabaqabeGadaaakeaacqWGHbqydaqhaaWcbaGaeG4mamdabaGaeGymaedaaaaa@3008@ = 1) and (a02
 MathType@MTEF@5@5@+=feaafiart1ev1aaatCvAUfKttLearuWrP9MDH5MBPbIqV92AaeXatLxBI9gBaebbnrfifHhDYfgasaacH8akY=wiFfYdH8Gipec8Eeeu0xXdbba9frFj0=OqFfea0dXdd9vqai=hGuQ8kuc9pgc9s8qqaq=dirpe0xb9q8qiLsFr0=vr0=vr0dc8meaabaqaciaacaGaaeqabaqabeGadaaakeaacqWGHbqydaqhaaWcbaGaeGimaadabaGaeGOmaidaaaaa@3004@ = 0, a12
 MathType@MTEF@5@5@+=feaafiart1ev1aaatCvAUfKttLearuWrP9MDH5MBPbIqV92AaeXatLxBI9gBaebbnrfifHhDYfgasaacH8akY=wiFfYdH8Gipec8Eeeu0xXdbba9frFj0=OqFfea0dXdd9vqai=hGuQ8kuc9pgc9s8qqaq=dirpe0xb9q8qiLsFr0=vr0=vr0dc8meaabaqaciaacaGaaeqabaqabeGadaaakeaacqWGHbqydaqhaaWcbaGaeGymaedabaGaeGOmaidaaaaa@3006@ = 100^1/0.7^, a22
 MathType@MTEF@5@5@+=feaafiart1ev1aaatCvAUfKttLearuWrP9MDH5MBPbIqV92AaeXatLxBI9gBaebbnrfifHhDYfgasaacH8akY=wiFfYdH8Gipec8Eeeu0xXdbba9frFj0=OqFfea0dXdd9vqai=hGuQ8kuc9pgc9s8qqaq=dirpe0xb9q8qiLsFr0=vr0=vr0dc8meaabaqaciaacaGaaeqabaqabeGadaaakeaacqWGHbqydaqhaaWcbaGaeGOmaidabaGaeGOmaidaaaaa@3008@ = -0.7, a32
 MathType@MTEF@5@5@+=feaafiart1ev1aaatCvAUfKttLearuWrP9MDH5MBPbIqV92AaeXatLxBI9gBaebbnrfifHhDYfgasaacH8akY=wiFfYdH8Gipec8Eeeu0xXdbba9frFj0=OqFfea0dXdd9vqai=hGuQ8kuc9pgc9s8qqaq=dirpe0xb9q8qiLsFr0=vr0=vr0dc8meaabaqaciaacaGaaeqabaqabeGadaaakeaacqWGHbqydaqhaaWcbaGaeG4mamdabaGaeGOmaidaaaaa@300A@ = 1) for mixed shape. Gene expression was generated by an exponential distribution with parameter *λ *= 1/3000 and the outliers were generated by a Beta distribution with parameters *B*(1.7,4.8). For more details, see Balagurunathan et al. (2002).

**Figure 1:**
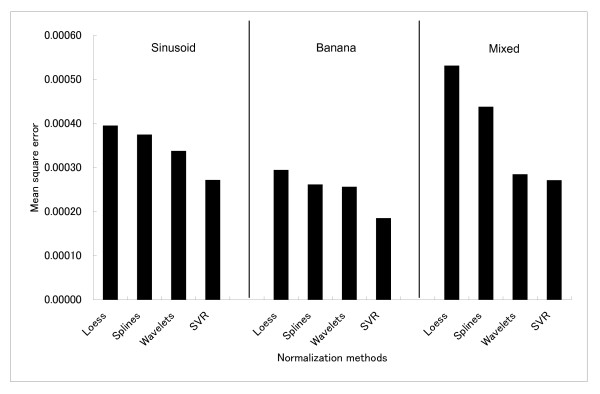
The minimum mean square error for three different simulated microarray datasets. From left to right: 1) sinusoid shape; 2) banana shape; 3) mix shape. The Kernel regression was not included in this figure because its MSE is 10^3 ^orders of magnitude greater than the other normalization methods.

The smoothing parameters used in each dataset are described in Table 1. For SVR, we tested a range of values and, as a result, we selected *ε *= 0.01 and *C *= 4 as the most adequate one. It is important to highlight that the parameters are arbitrary; therefore, we chose the optimum parameters for each method, i.e., the one which resulted in the lowest mean square error. In Figure 1 are described the mean square errors for each normalization method applied to three different simulated microarrays with no outliers.

**Table 1:** Smoothing parameters used for each microarray dataset. For Loess, it is the span value, for Splines and Wavelets it is the number of functions, for Kernel and SVR it is the maximum value minus the minimum value multiplied by the number described in the table.

	**Banana**	**Sinusoid**	**Mixed**
**Loess**	0.30	0.10	0.10
**Splines**	10.00	20.00	20.00
**Wavelets**	16.00	64.00	16.00
**Kernel**	0.50	0.50	0.70
**SVR**	0.20	0.20	0.60

In order to compare the perturbation caused by the presence of outliers and the robustness of each normalization method, we randomly inserted 5, 10, 15, 20 and 40% of outliers (genes which display very high differential expression) at three different expression levels (low, medium, high), and the respective mean square errors between the regression curve and the actual curve (the function from which the microarray was generated) was calculated. This step was repeated 100 times to estimate the average sum of the squared errors and their variance. The Wilcoxon and the Kolmogorov-Smirnov tests were performed in order to determine whether the five regression methods differ from one another in any significant manner.

A high performance normalization technique should yield unbiased corrections and corrections with the smallest standard deviation.

Comparison of the results presented in Table 2, 3 and 4 shows no important difference between LR, SS and WS. Although the non-parametric KR method has been successfully applied in econometrics data analysis [22], it displayed a poor performance for microarray normalization, probably because it is highly sensitive to outliers [23].

**Table 2:** The mean square errors of estimated gene expression levels for simulated cDNA microarray data with differentially expressed genes inserted under the low expression levels condition.

Percentage of DEG		Sinusoid			Banana			Mixed		
	
	Method	25% Quantile	Median	75% Quantile	25% Quantile	Median	75% Quantile	25% Quantile	Median	75% Quantile
5%	Loess	0.00038	0.00039	0.00040	0.00029	0.00029	0.00029	0.04806	0.04816	0.04825
	Splines	0.00035	0.00036	0.00037	0.00027	0.00028	0.00028	0.04828	0.04839	0.04848
	Wavelets	0.00033	0.00034	0.00035	0.00127	0.00128	0.00128	0.04816	0.04827	0.04835
	Kernel	0.03781	0.03782	0.03783	0.14368	0.14404	0.14416	0.19869	0.19888	0.20098
	SVR	0.00031	0.00032	0.00033	0.00016	0.00016	0.00017	0.04729	0.04733	0.04738
										
10%	Loess	0.00047	0.00048	0.00050	0.00038	0.00038	0.00039	0.04631	0.04646	0.04661
	Splines	0.00044	0.00045	0.00047	0.00037	0.00038	0.00039	0.04649	0.04663	0.04678
	Wavelets	0.00042	0.00043	0.00045	0.00117	0.00118	0.00118	0.04637	0.04651	0.04666
	Kernel	0.03780	0.03781	0.03783	0.15535	0.15574	0.15602	0.19177	0.19337	0.19596
	SVR	0.00040	0.00041	0.00043	0.00031	0.00032	0.00033	0.04543	0.04548	0.04556
										
15%	Loess	0.00055	0.00057	0.00059	0.00037	0.00038	0.00040	0.05157	0.05177	0.05194
	Splines	0.00053	0.00055	0.00057	0.00036	0.00036	0.00038	0.05180	0.05199	0.05215
	Wavelets	0.00050	0.00053	0.00054	0.00085	0.00087	0.00088	0.05165	0.05184	0.05199
	Kernel	0.03779	0.03781	0.03784	0.16922	0.16965	0.17000	0.18852	0.18989	0.19142
	SVR	0.00048	0.00050	0.00052	0.00033	0.00034	0.00035	0.05057	0.05069	0.05078
										
20%	Loess	0.00064	0.00066	0.00068	0.00042	0.00043	0.00044	0.04780	0.04797	0.04819
	Splines	0.00061	0.00063	0.00066	0.00040	0.00042	0.00043	0.04799	0.04818	0.04837
	Wavelets	0.00059	0.00061	0.00064	0.00138	0.00140	0.00142	0.04786	0.04807	0.04825
	Kernel	0.03778	0.03781	0.03785	0.14796	0.14841	0.14864	0.18435	0.18606	0.18721
	SVR	0.00056	0.00058	0.00060	0.00035	0.00036	0.00037	0.04630	0.04638	0.04647
										
40%	Loess	0.00098	0.00102	0.00104	0.00057	0.00061	0.00065	0.07937	0.07985	0.08031
	Splines	0.00096	0.00099	0.00103	0.00060	0.00064	0.00069	0.07965	0.08014	0.08059
	Wavelets	0.00095	0.00098	0.00101	0.00178	0.00182	0.00187	0.07954	0.08003	0.08047
	Kernel	0.03771	0.03780	0.03786	0.14208	0.14235	0.14271	0.20321	0.20363	0.20426
	SVR	0.00088	0.00091	0.00094	0.00047	0.00048	0.00049	0.06863	0.06901	0.06943

DEG: Differentially expressed genes

**Table 3:** The mean square errors of estimated gene expression levels for simulated cDNA microarray data with differentially expressed genes inserted under the medium expression levels condition.

Percentage of DEG		Sinusoid			Banana			Mixed		
	
	Method	25% Quantile	Median	75% Quantile	25% Quantile	Median	75% Quantile	25% Quantile	Median	75% Quantile
5%	Loess	0.00356	0.00373	0.00389	0.00379	0.00392	0.00407	0.05214	0.05234	0.05259
	Splines	0.00354	0.00370	0.00387	0.00379	0.00393	0.00407	0.05237	0.05258	0.05283
	Wavelets	0.00351	0.00368	0.00384	0.00438	0.00450	0.00466	0.05227	0.05247	0.05272
	Kernel	0.03799	0.03816	0.03838	0.17380	0.17441	0.17487	0.18858	0.18882	0.18904
	SVR	0.00337	0.00353	0.00369	0.00357	0.00368	0.00382	0.05034	0.05049	0.05067
										
10%	Loess	0.00709	0.00723	0.00743	0.00758	0.00780	0.00799	0.05483	0.05506	0.05532
	Splines	0.00707	0.00721	0.00741	0.00763	0.00787	0.00805	0.05507	0.05532	0.05556
	Wavelets	0.00705	0.00718	0.00739	0.00858	0.00881	0.00898	0.05497	0.05522	0.05547
	Kernel	0.03837	0.03857	0.03886	0.15366	0.15461	0.15523	0.18801	0.18830	0.18867
	SVR	0.00672	0.00688	0.00707	0.00709	0.00731	0.00750	0.05150	0.05164	0.05183
										
15%	Loess	0.01041	0.01061	0.01094	0.01108	0.01136	0.01165	0.05964	0.05985	0.06012
	Splines	0.01039	0.01060	0.01091	0.01109	0.01136	0.01165	0.05990	0.06013	0.06039
	Wavelets	0.01038	0.01058	0.01089	0.01251	0.01276	0.01310	0.05978	0.06003	0.06029
	Kernel	0.03867	0.03897	0.03927	0.12923	0.13026	0.13111	0.19337	0.19367	0.19414
	SVR	0.00986	0.01006	0.01032	0.01027	0.01056	0.01081	0.05499	0.05526	0.05550
										
20%	Loess	0.01393	0.01418	0.01444	0.01487	0.01519	0.01542	0.06362	0.06398	0.06432
	Splines	0.01393	0.01415	0.01442	0.01486	0.01518	0.01542	0.06390	0.06425	0.06460
	Wavelets	0.01390	0.01414	0.01440	0.01631	0.01666	0.01689	0.06375	0.06410	0.06445
	Kernel	0.03915	0.03957	0.04004	0.12265	0.12366	0.12464	0.19808	0.19858	0.19909
	SVR	0.01310	0.01334	0.01365	0.01365	0.01399	0.01416	0.05809	0.05835	0.05858
										
40%	Loess	0.02772	0.02813	0.02862	0.02969	0.03004	0.03043	0.07856	0.07910	0.07975
	Splines	0.02774	0.02814	0.02861	0.02966	0.03002	0.03038	0.07884	0.07937	0.08002
	Wavelets	0.02771	0.02811	0.02859	0.03012	0.03049	0.03092	0.07873	0.07926	0.07995
	Kernel	0.04195	0.04261	0.04316	0.15640	0.15816	0.16012	0.20368	0.20443	0.20518
	SVR	0.02545	0.02581	0.02614	0.02656	0.02685	0.02724	0.06786	0.06830	0.06862

DEG: Differentially expressed genes

**Table 4:** The mean square errors of estimated gene expression levels for simulated cDNA microarray data with differentially expressed genes inserted under the high expression levels conditions.

Percentage of DEG		Sinusoid			Banana			Mixed		
	
	Method	25% Quantile	Median	75% Quantile	25% Quantile	Median	75% Quantile	25% Quantile	Median	75% Quantile
5%	Loess	0.00038	0.00039	0.00040	0.00081	0.00087	0.00094	0.04633	0.04639	0.04648
	Splines	0.00035	0.00036	0.00037	0.00079	0.00086	0.00092	0.04658	0.04665	0.04674
	Wavelets	0.00033	0.00034	0.00035	0.00115	0.00121	0.00129	0.04643	0.04650	0.04660
	Kernel	0.03781	0.03782	0.03783	0.16417	0.16453	0.16513	0.17428	0.17436	0.17447
	SVR	0.00031	0.00032	0.00033	0.00080	0.00087	0.00092	0.04527	0.04534	0.04544
										
10%	Loess	0.00146	0.00153	0.00168	0.00145	0.00156	0.00167	0.04733	0.04748	0.04758
	Splines	0.00142	0.00149	0.00160	0.00147	0.00157	0.00168	0.04756	0.04767	0.04779
	Wavelets	0.00140	0.00147	0.00159	0.00160	0.00170	0.00182	0.04725	0.04736	0.04748
	Kernel	0.02662	0.03454	0.03789	0.23086	0.23135	0.23245	0.18441	0.19003	0.19176
	SVR	0.00126	0.00133	0.00144	0.00142	0.00154	0.00166	0.04678	0.04687	0.04696
										
15%	Loess	0.00203	0.00217	0.00234	0.00199	0.00212	0.00224	0.04963	0.04976	0.04991
	Splines	0.00198	0.00211	0.00223	0.00200	0.00211	0.00222	0.04987	0.05001	0.05014
	Wavelets	0.00196	0.00209	0.00224	0.00219	0.00240	0.00257	0.04975	0.04989	0.05001
	Kernel	0.02318	0.02992	0.03729	0.17578	0.20066	0.22763	0.18472	0.18898	0.18923
	SVR	0.00178	0.00190	0.00200	0.00170	0.00180	0.00189	0.04885	0.04898	0.04912
										
20%	Loess	0.00260	0.00275	0.00293	0.00259	0.00272	0.00289	0.04917	0.04930	0.04944
	Splines	0.00254	0.00268	0.00286	0.00260	0.00270	0.00289	0.04933	0.04946	0.04961
	Wavelets	0.00253	0.00267	0.00284	0.00289	0.00304	0.00320	0.04919	0.04933	0.04947
	Kernel	0.02224	0.02819	0.03468	0.16500	0.17817	0.20385	0.18207	0.18716	0.19141
	SVR	0.00226	0.00239	0.00255	0.00247	0.00258	0.00272	0.04839	0.04850	0.04863
										
40%	Loess	0.00501	0.00520	0.00545	0.00518	0.00538	0.00555	0.04980	0.04999	0.05020
	Splines	0.00498	0.00519	0.00539	0.00520	0.00538	0.00558	0.05002	0.05022	0.05038
	Wavelets	0.00496	0.00517	0.00537	0.00535	0.00551	0.00572	0.04984	0.05004	0.05020
	Kernel	0.02155	0.02487	0.02810	0.18250	0.20140	0.22296	0.17524	0.18072	0.18433
	SVR	0.00446	0.00467	0.00489	0.00464	0.00483	0.00505	0.04809	0.04829	0.04860

DEG: Differentially expressed genes

Upon analyzing Table 2, it is possible to observe, in the case of sinusoid shape, that when outliers are inserted in regions of low gene expression, SVR, WS, SS, LR and KR, in this order, have the lowest to the highest mean square error, being statistically different (p value < 0.001) from one another. For the banana and mixed shapes, LR and SS presented a lower MSE than WS. In Table 3, it is interesting to note that when outliers are inserted in regions of medium gene expression, i.e., high density of genes, the order of performance remains the same as in Table 2 and SVR displays a mean square error which is significantly different from the others (p value < 0.001). LR and SS showed no significant difference (p value > 0.05) and KR is significantly worse than the other methods (p value < 0.001). In Table 4, the outliers are inserted in a high gene expression region. Once more, the trend is maintained, namely, KR is the most affected by outliers (p value < 0.001) and no differences between SS and WS (p value > 0.05) were observed for the sinusoid shape. For the other two shapes, LR and SS were better than WS (p value < 0.001).

In all three cases (outliers at low, medium and high gene expression), SVR is the affected by outliers (p value < 0.001), independently the microarray's shape. In addition, SVR yields the smallest standard deviation, followed by LR, SS, WS, with KR displaying the largest deviation. In addition, the five methods were applied to actual microarray data, with outliers inserted artificially, and the results were the same when compared to those obtained from artificial microarray experiments.

**Figure 2:**
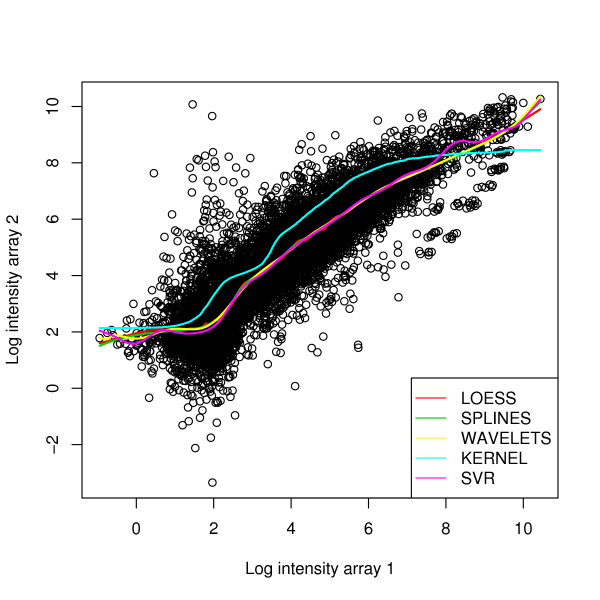
Fitted normalization curves for actual cDNA microarray data using the five different normalization methods (Loess, Splines, Wavelets, Kernel, SVR).

In Figure 2, we illustrate the performance of the five normalization methods applied to actual microarray data, without the insertion of artificial outliers. A small difference could be observed in the normalization curves in which the genes displayed low and high expression, due to the low quantity of genes and the high variance.

## Discussion

By analyzing the extent to which the outliers could disturb the regression curve, we observed that KR is more highly sensitive to outliers than LR, SS and WS in all three cases (outliers in low, medium and high expression). In all three cases, SVR is shown to be the least affected.

The superior performance of Splines, when compared to KR, may be explained by the degree of smoothing, which varies according to the density of points, differently from KR, which has a fixed window size. Wavelet also has a slightly better performance than KR, probably due to multi-resolution properties. In general, SS and WS presented similar performance when we compared the median of the mean square error using the Wilcoxon test. However, when we used the Kolmogorov-Smirnov test, they presented a statistically significant difference (p value < 0.001). SS and WS constitute somewhat better normalization techniques than LR when we analyzed the sinusoid shape, but, for the other two shapes, LR is better than SS and WS. For practical purposes, the differences between them in terms of disturbance by outliers are too small to be of any concern.

The SVR method is shown to be very robust to outliers presented at different gene expression levels, becoming the best normalization technique to identify actual differentially expressed genes.

One well-known problem in identifying differentially expressed genes is normalizing genes displaying low expression levels, due to the low quantity of the corresponding transcripts and the high spot intensity variance. An equivalent problem occurs with genes presenting very high expression levels due to the low frequency of these genes. Once more, under these conditions, the SVR method is shown to be better than other currently used methods.

We performed the same tests for five other pairs of CodeLink™ microarrays and the results obtained were the same: the SVR is the most robust to outliers and the KR method is the worst method, being highly sensitive to differentially expressed genes and yielding poor regression curves.

Other methods, which are also robust to outliers and are based on a new regression method called two-way semi-linear model [24-27] have also been applied for microarray data normalization. This new approach developed in the last few years, deserves further studies, which we are planning to undertake in the future.

## Conclusion

We have proposed a new approach to normalize microarray data and tested this SVR method by benchmark studies and by several simulations. The results obtained with SVR were superior than those obtained with some widely used normalization techniques such as LR, SS and WS. SVR is shown to be more robust to outliers even at very low and very high gene expression levels, being useful to identify differentially expressed genes. Even tested in different microarray shapes, SVR was superior to the other methods, while LR, SS and WS presented similar performances. Therefore, we have demonstrated that SVR is feasible and very promising for microarray data normalization.

## Methods

### Simulation

The program which generates the artificial microarray and the analyses were implemented in R, a language for statistical computing [28]. This script may be downloaded at: [29].

### CodeLink™ microarray

#### Cell lysis and RNA extraction

Cell cultures were lysed with guanidine isocyanate and RNA was purified by of the cell lysates on a cesium chloride cushion (Chirgwin el al, 1979). Absorbance ratio at 260/280 nm was used to assess the RNA purity, a ratio of 1.8 – 2.0 indicating adequate purity.

#### Labeling and purification of targets

RNA samples were prepared and processed according to protocols supplied by the manufacturer (Amersham Biosciences). Briefly, cDNAs were synthesized from purified RNA (2 μg) and control bacterial mRNAs. Samples were purified using the QIAquick Spin kit (Qiagen) and concentrated by SpeedVac. Concentrated pellets were used in a biotinylated-UTP based cRNA synthesis using the CodeLink™ Expression Assay Reagent Kit (Amersham). Labeled cRNAs were purified using the RNeasy kit (Qiagen) and fragmented with supplied solution at 94°C for 20 min.

#### Hybridization and washing of arrays

Fragmented biotin-labeled cRNAs (10 μg) were incubated with CodeLink™ bioarrays and shaken (300 rpm) for 20 h. The bioarrays were then washed and incubated with Cy5-Streptavidin (30 min). Scanning of the bioarrays was performed in a GenePix 4000 B Array Scanner (Axon Instruments) and the data were collected using the CodeLink™ System Software (Amersham), which provided the raw data and invalidated data from irregular spots.

### Loess regression

Consider we have *n *measurements, for each of which the response expected is *y*_*i *_and let *x*_*i *_be the predictor, where *x *is the log intensity of one microarray and *y *is the log intensity of the other one, in case we are analyzing a single-color microarrays. Whether the microarray is a two-color platform, *x *is the log of one dye intensity and *y *is the log of the other dye intensity.

In this model, they are supposed to be related by

*y*_*i *_= *g*(*x*_*i*_) + *ε*_*i *_    (1)

where *g *is the regression function and *ε*_*i *_is a random error. The idea of local regression is that near *x *= *x*_0_, the regression function *g*(*x*) can be locally approximated by the value of a function in some specified parametric class. Such a local approximation is obtained by fitting a regression surface to the data points within a chosen neighborhood of the point *x*_0_.

In this method, weighed least squares are used to fit linear or quadratic functions of the predictors at the centers of the neighborhoods. The radius of each neighborhood is chosen so that the neighborhood contains a specified percentage of the data points. The fraction of the data, called the smoothing parameter, in each local neighborhood is weighted by a smooth decreasing function of their distance from the center of the neighborhood [30].

### B-Splines smoothing

Due to its simple structure and good approximation properties, polynomials are widely used in practice for approximating functions [31,32]. Let *x *and *y *as defined above and

(x−y)+0={1,x≥y0,x<y     (2)
 MathType@MTEF@5@5@+=feaafiart1ev1aaatCvAUfKttLearuWrP9MDH5MBPbIqV92AaeXatLxBI9gBaebbnrfifHhDYfgasaacH8akY=wiFfYdH8Gipec8Eeeu0xXdbba9frFj0=OqFfea0dXdd9vqai=hGuQ8kuc9pgc9s8qqaq=dirpe0xb9q8qiLsFr0=vr0=vr0dc8meaabaqaciaacaGaaeqabaqabeGadaaakeaacqGGOaakcqWG4baEcqGHsislcqWG5bqEcqGGPaqkdaqhaaWcbaGaey4kaScabaGaeGimaadaaOGaeyypa0ZaaiqabeaafaqabeGabaaabaGaeGymaeJaeiilaWIaemiEaGNaeyyzImRaemyEaKhabaGaeGimaaJaeiilaWIaemiEaGNaeyipaWJaemyEaKhaaaGaay5EaaGaaCzcaiaaxMaadaqadaqaaiabikdaYaGaayjkaiaawMcaaaaa@4683@

and

(x−y)+m−1={(x−y)m−1,x≥y,m>10,x<y     (3)
 MathType@MTEF@5@5@+=feaafiart1ev1aaatCvAUfKttLearuWrP9MDH5MBPbIqV92AaeXatLxBI9gBaebbnrfifHhDYfgasaacH8akY=wiFfYdH8Gipec8Eeeu0xXdbba9frFj0=OqFfea0dXdd9vqai=hGuQ8kuc9pgc9s8qqaq=dirpe0xb9q8qiLsFr0=vr0=vr0dc8meaabaqaciaacaGaaeqabaqabeGadaaakeaacqGGOaakcqWG4baEcqGHsislcqWG5bqEcqGGPaqkdaqhaaWcbaGaey4kaScabaGaemyBa0MaeyOeI0IaeGymaedaaOGaeyypa0ZaaiqabeaafaqaaeGabaaabaGaeiikaGIaemiEaGNaeyOeI0IaemyEaKNaeiykaKYaaWbaaSqabeaacqWGTbqBcqGHsislcqaIXaqmaaGccqGGSaalcqWG4baEcqGHLjYScqWG5bqEcqGGSaalcqWGTbqBcqGH+aGpcqaIXaqmaeaacqaIWaamcqGGSaalcqWG4baEcqGH8aapcqWG5bqEaaaacaGL7baacaWLjaGaaCzcaiabcIcaOiabiodaZiabcMcaPaaa@5554@

Therefore, let

... ≤ *y*_-1 _≤ *y*_0 _≤ *y*_1 _≤ *y*_2 _≤ ...     (4)

be a sequence of real numbers. Given integers *i *and *m *> 0, we define

Qim(x)={(−1)m[yi,...,yi+m](x−y)+m−1,if  yi<yi+m0,otherwise     (5)
 MathType@MTEF@5@5@+=feaafiart1ev1aaatCvAUfKttLearuWrP9MDH5MBPbIqV92AaeXatLxBI9gBaebbnrfifHhDYfgasaacH8akY=wiFfYdH8Gipec8Eeeu0xXdbba9frFj0=OqFfea0dXdd9vqai=hGuQ8kuc9pgc9s8qqaq=dirpe0xb9q8qiLsFr0=vr0=vr0dc8meaabaqaciaacaGaaeqabaqabeGadaaakeaacqWGrbqudaqhaaWcbaGaemyAaKgabaGaemyBa0gaaOGaeiikaGIaemiEaGNaeiykaKIaeyypa0ZaaiqabeaafaqaaeGabaaabaGaeiikaGIaeyOeI0IaeGymaeJaeiykaKYaaWbaaSqabeaacqWGTbqBaaGccqGGBbWwcqWG5bqEdaWgaaWcbaGaemyAaKgabeaakiabcYcaSiabc6caUiabc6caUiabc6caUiabcYcaSiabdMha5naaBaaaleaacqWGPbqAcqGHRaWkcqWGTbqBaeqaaOGaeiyxa0LaeiikaGIaemiEaGNaeyOeI0IaemyEaKNaeiykaKYaa0baaSqaaiabgUcaRaqaaiabd2gaTjabgkHiTiabigdaXaaakiabcYcaSiabdMgaPjabdAgaMjaaykW7caaMc8UaemyEaK3aaSbaaSqaaiabdMgaPbqabaGccqGH8aapcqWG5bqEdaWgaaWcbaGaemyAaKMaey4kaSIaemyBa0gabeaaaOqaaiabicdaWiabcYcaSiabd+gaVjabdsha0jabdIgaOjabdwgaLjabdkhaYjabdEha3jabdMgaPjabdohaZjabdwgaLbaaaiaawUhaaiaaxMaacaWLjaGaeiikaGIaeGynauJaeiykaKcaaa@76CA@

for all real *x*. We call Qim
 MathType@MTEF@5@5@+=feaafiart1ev1aaatCvAUfKttLearuWrP9MDH5MBPbIqV92AaeXatLxBI9gBaebbnrfifHhDYfgasaacH8akY=wiFfYdH8Gipec8Eeeu0xXdbba9frFj0=OqFfea0dXdd9vqai=hGuQ8kuc9pgc9s8qqaq=dirpe0xb9q8qiLsFr0=vr0=vr0dc8meaabaqaciaacaGaaeqabaqabeGadaaakeaacqWGrbqudaqhaaWcbaGaemyAaKgabaGaemyBa0gaaaaa@30C2@ the *m *th order B-Spline associated with the knots *y*_*i *_,..., *y*_*i *+ *m*_.

For *m *= 1, the B-Spline associated with *y*_*i *_<*y*_*i *+ 1 _is particularly simple. It is the piecewise constant function

Qi1(x)={1yi+1−yi,yi≤x≤yi+10,otherwise.     (6)
 MathType@MTEF@5@5@+=feaafiart1ev1aaatCvAUfKttLearuWrP9MDH5MBPbIqV92AaeXatLxBI9gBaebbnrfifHhDYfgasaacH8akY=wiFfYdH8Gipec8Eeeu0xXdbba9frFj0=OqFfea0dXdd9vqai=hGuQ8kuc9pgc9s8qqaq=dirpe0xb9q8qiLsFr0=vr0=vr0dc8meaabaqaciaacaGaaeqabaqabeGadaaakeaacqWGrbqudaqhaaWcbaGaemyAaKgabaGaeGymaedaaOGaeiikaGIaemiEaGNaeiykaKIaeyypa0ZaaiqabeaafaqaaeGabaaabaWaaSaaaeaacqaIXaqmaeaacqWG5bqEdaWgaaWcbaGaemyAaKMaey4kaSIaeGymaedabeaakiabgkHiTiabdMha5naaBaaaleaacqWGPbqAaeqaaaaakiabcYcaSiabdMha5naaBaaaleaacqWGPbqAaeqaaOGaeyizImQaemiEaGNaeyizImQaemyEaK3aaSbaaSqaaiabdMgaPjabgUcaRiabigdaXaqabaaakeaacqaIWaamcqGGSaalcqWGVbWBcqWG0baDcqWGObaAcqWGLbqzcqWGYbGCcqWG3bWDcqWGPbqAcqWGZbWCcqWGLbqzcqGGUaGlaaaacaGL7baacaWLjaGaaCzcaiabcIcaOiabiAda2iabcMcaPaaa@605C@

In our analysis, we applied the cubic Splines, i.e., Splines of order 3.

We can also give explicit formulate for Qim
 MathType@MTEF@5@5@+=feaafiart1ev1aaatCvAUfKttLearuWrP9MDH5MBPbIqV92AaeXatLxBI9gBaebbnrfifHhDYfgasaacH8akY=wiFfYdH8Gipec8Eeeu0xXdbba9frFj0=OqFfea0dXdd9vqai=hGuQ8kuc9pgc9s8qqaq=dirpe0xb9q8qiLsFr0=vr0=vr0dc8meaabaqaciaacaGaaeqabaqabeGadaaakeaacqWGrbqudaqhaaWcbaGaemyAaKgabaGaemyBa0gaaaaa@30C2@ in case either *y*_*i *_or *y*_*i *+ *m *_is a knot of multiplicity *m*.

### Wavelet smoothing

The Wavelet transform is a relatively new approach and has some similarities with the Fourier transform. Wavelets differ from Fourier methods in that they allow the localization of a signal in both time and frequency. In the wavelet theory, a function is represented by an infinite series expansion in terms of dilated and translated version of a basic function *ψ *called the "mother' Wavelet. A Wavelet transformation leads to an additive decomposition of a signal into a series of different components describing smooth and rough features of the signal.

The term Wavelets means small curves, therefore, they are oscillations that rapidly decay. As the B-Splines functions system, the Wavelets functions *ψ*(*t*) can be used to generate a function basis for certain spaces [33]. An ortonormal basis can be generated by dyadic dilations and translations of a mother Wavelet *ψ*(*t*), by

*ψ*_*j, k *_(*t*) = 2^*j*/2 ^*ψ *(2^*j *^*j *- *k*),     *j*, *k *∈ Z     (7)

Wavelets are functions which satisfy the following properties:

i) ∫−∞∞ψ(t)
MathType@MTEF@5@5@+=feaafiart1ev1aaatCvAUfKttLearuWrP9MDH5MBPbIqV92AaeXatLxBI9gBaebbnrfifHhDYfgasaacH8akY=wiFfYdH8Gipec8Eeeu0xXdbba9frFj0=OqFfea0dXdd9vqai=hGuQ8kuc9pgc9s8qqaq=dirpe0xb9q8qiLsFr0=vr0=vr0dc8meaabaqaciaacaGaaeqabaqabeGadaaakeaadaWdXbqaaGGaciab=H8a5bWcbaGaeyOeI0IaeyOhIukabaGaeyOhIukaniabgUIiYdGccqGGOaakcqWG0baDcqGGPaqkaaa@37E6@*dt *= 0     (8).

ii) ∫−∞∞ψ(t)
MathType@MTEF@5@5@+=feaafiart1ev1aaatCvAUfKttLearuWrP9MDH5MBPbIqV92AaeXatLxBI9gBaebbnrfifHhDYfgasaacH8akY=wiFfYdH8Gipec8Eeeu0xXdbba9frFj0=OqFfea0dXdd9vqai=hGuQ8kuc9pgc9s8qqaq=dirpe0xb9q8qiLsFr0=vr0=vr0dc8meaabaqaciaacaGaaeqabaqabeGadaaakeaadaWdXbqaaGGaciab=H8a5naabmaabaGaemiDaqhacaGLOaGaayzkaaaaleaacqGHsislcqGHEisPaeaacqGHEisPa0Gaey4kIipaaaa@37B3@*dt *< ∞     (9).

iii) ∫−∞∞|Ψ(ω)|2dω|ω|=0
MathType@MTEF@5@5@+=feaafiart1ev1aaatCvAUfKttLearuWrP9MDH5MBPbIqV92AaeXatLxBI9gBaebbnrfifHhDYfgasaacH8akY=wiFfYdH8Gipec8Eeeu0xXdbba9frFj0=OqFfea0dXdd9vqai=hGuQ8kuc9pgc9s8qqaq=dirpe0xb9q8qiLsFr0=vr0=vr0dc8meaabaqaciaacaGaaeqabaqabeGadaaakeaadaWdXbqaamaalaaabaWaaqWaaeaacqqHOoqwcqGGOaakcqaHjpWDcqGGPaqkaiaawEa7caGLiWoadaahaaWcbeqaaiabikdaYaaakiabdsgaKjabeM8a3bqaamaaemaabaGaeqyYdChacaGLhWUaayjcSdaaaaWcbaGaeyOeI0IaeyOhIukabaGaeyOhIukaniabgUIiYdGccqGH9aqpcqaIWaamaaa@4658@, where the function Ψ(*ω*) is the Fourier transform of *ψ*(*t*)     (10).

iv) ∫−∞∞tjψ(t)dt=0
MathType@MTEF@5@5@+=feaafiart1ev1aaatCvAUfKttLearuWrP9MDH5MBPbIqV92AaeXatLxBI9gBaebbnrfifHhDYfgasaacH8akY=wiFfYdH8Gipec8Eeeu0xXdbba9frFj0=OqFfea0dXdd9vqai=hGuQ8kuc9pgc9s8qqaq=dirpe0xb9q8qiLsFr0=vr0=vr0dc8meaabaqaciaacaGaaeqabaqabeGadaaakeaadaWdXbqaaiabdsha0naaCaaaleqabaGaemOAaOgaaGGacOGae8hYdKNaeiikaGIaemiDaqNaeiykaKIaemizaqMaemiDaqNaeyypa0JaeGimaadaleaacqGHsislcqGHEisPaeaacqGHEisPa0Gaey4kIipaaaa@3F97@,     *j *= 0,1,..., *r *- 1     for *r *≥ 1 and ∫−∞∞|trψ(t)|dt<∞
MathType@MTEF@5@5@+=feaafiart1ev1aaatCvAUfKttLearuWrP9MDH5MBPbIqV92AaeXatLxBI9gBaebbnrfifHhDYfgasaacH8akY=wiFfYdH8Gipec8Eeeu0xXdbba9frFj0=OqFfea0dXdd9vqai=hGuQ8kuc9pgc9s8qqaq=dirpe0xb9q8qiLsFr0=vr0=vr0dc8meaabaqaciaacaGaaeqabaqabeGadaaakeaadaWdXbqaamaaemaabaGaemiDaq3aaWbaaSqabeaacqWGYbGCaaacciGccqWFipqEcqGGOaakcqWG0baDcqGGPaqkaiaawEa7caGLiWoaaSqaaiabgkHiTiabg6HiLcqaaiabg6HiLcqdcqGHRiI8aOGaemizaqMaemiDaqNaeyipaWJaeyOhIukaaa@4354@     (11).

An important result is that any function *f *(*t*) with ∫−∞∞f2(t)<∞
MathType@MTEF@5@5@+=feaafiart1ev1aaatCvAUfKttLearuWrP9MDH5MBPbIqV92AaeXatLxBI9gBaebbnrfifHhDYfgasaacH8akY=wiFfYdH8Gipec8Eeeu0xXdbba9frFj0=OqFfea0dXdd9vqai=hGuQ8kuc9pgc9s8qqaq=dirpe0xb9q8qiLsFr0=vr0=vr0dc8meaabaqaciaacaGaaeqabaqabeGadaaakeaadaWdXbqaaiabdAgaMnaaCaaaleqabaGaeGOmaidaaOGaeiikaGIaemiDaqNaeiykaKIaeyipaWJaeyOhIukaleaacqGHsislcqGHEisPaeaacqGHEisPa0Gaey4kIipaaaa@3AFA@ can be expanded as

f(t)=∑j=−∞∞∑k=−∞∞cj,kψj,k(t)     (12).
 MathType@MTEF@5@5@+=feaafiart1ev1aaatCvAUfKttLearuWrP9MDH5MBPbIqV92AaeXatLxBI9gBaebbnrfifHhDYfgasaacH8akY=wiFfYdH8Gipec8Eeeu0xXdbba9frFj0=OqFfea0dXdd9vqai=hGuQ8kuc9pgc9s8qqaq=dirpe0xb9q8qiLsFr0=vr0=vr0dc8meaabaqaciaacaGaaeqabaqabeGadaaakeaacqWGMbGzcqGGOaakcqWG0baDcqGGPaqkcqGH9aqpdaaeWbqaamaaqahabaGaem4yam2aaSbaaSqaaiabdQgaQjabcYcaSiabdUgaRbqabaaabaGaem4AaSMaeyypa0JaeyOeI0IaeyOhIukabaGaeyOhIukaniabggHiLdacciGccqWFipqEdaWgaaWcbaGaemOAaOMaeiilaWIaem4AaSgabeaaaeaacqWGQbGAcqGH9aqpcqGHsislcqGHEisPaeaacqGHEisPa0GaeyyeIuoakiabcIcaOiabdsha0jabcMcaPiaaxMaacaWLjaGaeiikaGIaeGymaeJaeGOmaiJaeiykaKIaeiOla4caaa@56A5@

In other words, any function *f *(*t*) can be represented by a linear combination of functions *ψ*_*j, k *_(*t*). The smoothing procedure can be carried out by an approximation, choosing a maximum resolution *J *(*t*) for *j *=1,2,..., *J *(*t*) and *k *= 1,2,..., 2^*j*-1^. Here, we considered the Mexican hat Wavelet [34] defined by

ψ(t)=(1−t2)exp⁡(−t22)     (13)
 MathType@MTEF@5@5@+=feaafiart1ev1aaatCvAUfKttLearuWrP9MDH5MBPbIqV92AaeXatLxBI9gBaebbnrfifHhDYfgasaacH8akY=wiFfYdH8Gipec8Eeeu0xXdbba9frFj0=OqFfea0dXdd9vqai=hGuQ8kuc9pgc9s8qqaq=dirpe0xb9q8qiLsFr0=vr0=vr0dc8meaabaqaciaacaGaaeqabaqabeGadaaakeaaiiGacqWFipqEcqGGOaakcqWG0baDcqGGPaqkcqGH9aqpcqGGOaakcqaIXaqmcqGHsislcqWG0baDdaahaaWcbeqaaiabikdaYaaakiabcMcaPiGbcwgaLjabcIha4jabcchaWnaabmaabaWaaSaaaeaacqGHsislcqWG0baDdaahaaWcbeqaaiabikdaYaaaaOqaaiabikdaYaaaaiaawIcacaGLPaaacaWLjaGaaCzcaiabcIcaOiabigdaXiabiodaZiabcMcaPaaa@47F3@

rather than other functions such as Morlet or Shannon since they do not have an analytic formula.

The *C*_*jk *_coefficients are estimated via an ordinary least square regression. An important feature in the wavelets representation is that it allows the description of functions belonging to both Sobolev and Besov spaces [35].

### Kernel regression

KR is one class of modeling methods that belongs to the smoothing methods family. It is part of the non-parametric regression methods. KR allows basing the prediction of a value on passed observations, and weighing the impact of past observations depending on how similar they are, compared to the current values of the explanatory variables.

The KR is one of the most widely used procedures in non-parametric curve estimation. Nadaraya (1964) and Watson (1964) proposed an estimator for the curve *g *given by

gh(x)=∑i=1nKh(x−Xi)Yi∑j=1nKh(x−Xj)     (14)
 MathType@MTEF@5@5@+=feaafiart1ev1aaatCvAUfKttLearuWrP9MDH5MBPbIqV92AaeXatLxBI9gBaebbnrfifHhDYfgasaacH8akY=wiFfYdH8Gipec8Eeeu0xXdbba9frFj0=OqFfea0dXdd9vqai=hGuQ8kuc9pgc9s8qqaq=dirpe0xb9q8qiLsFr0=vr0=vr0dc8meaabaqaciaacaGaaeqabaqabeGadaaakeaacqWGNbWzdaWgaaWcbaGaemiAaGgabeaakiabcIcaOiabdIha4jabcMcaPiabg2da9maalaaabaWaaabCaeaacqWGlbWsdaWgaaWcbaGaemiAaGgabeaaaeaacqWGPbqAcqGH9aqpcqaIXaqmaeaacqWGUbGBa0GaeyyeIuoakiabcIcaOiabdIha4jabgkHiTiabdIfaynaaBaaaleaacqWGPbqAaeqaaOGaeiykaKIaemywaK1aaSbaaSqaaiabdMgaPbqabaaakeaadaaeWbqaaiabdUealnaaBaaaleaacqWGObaAaeqaaaqaaiabdQgaQjabg2da9iabigdaXaqaaiabd6gaUbqdcqGHris5aOGaeiikaGIaemiEaGNaeyOeI0IaemiwaG1aaSbaaSqaaiabdQgaQbqabaGccqGGPaqkaaGaaCzcaiaaxMaacqGGOaakcqaIXaqmcqaI0aancqGGPaqkaaa@5C79@

In our datasets, we used the Gaussian Kernel because it is symmetric and centralized in the mean.

In addition to being easy to compute, the Nadaraya-Watson estimator *g*_*h *_(*x*) is consistent. When *h *→ 0 the estimated curve presents a large variability and when *nh *→ ∞, we obtain an overly smooth curve [36]. The bandwidth *h *controls the smoothness degree of the estimated curve. It is easy to observe that this KR estimator is just a weighted sum of the observed responses *Y*_*i*_. The denominator ensures that the weights sum up to 1.

### Support Vector Regression

SVR generalized algorithm is a non-linear regression from the Generalized Portrait algorithm developed in Russia by Vapnik and Lerner (1963) [37] and Vapnik and Chervonenkis (1964) [38]. It is based upon the statistical learning theory which has been developed by Vapnik and Chervonenkis (1974) [39]. In Bioinformatics, and, more specifically, in microarray data analysis, to the best of our knowledge, this algorithm has previously been used only once, by Hisanori et al. (2004), to extract relations between promoter sequences and strengths [40]. Here, we propose the use of SVR to normalize microarray data.

Let {(*x*_1_, *y*_1_),..., (*x*_1_, *y*_1_)} ⊂ *R *× *R *be the gene expression data derived from microarray experiments, where *x *is the log intensity of one microarray and *y *is the log intensity of the other one, in case we are analyzing a single-color microarrays. When the microarray is a two-color platform, *x *is the log of one dye intensity and *y *is the log of the other dye intensity. In *ε*-SVR [41], the goal is to obtain a function *f *(*x*) that has at the most *ε *deviation from the *y*_*i *_for all the data, and is as flat as possible.

In the case of linear functions *f *:

*f *(*x*) = (*w*^*t *^*x*) + *b *with *w *∈ *R*^*n*^, *b *∈ *R *    (15)

Flatness in (15) means

Minimize 12‖w‖2
 MathType@MTEF@5@5@+=feaafiart1ev1aaatCvAUfKttLearuWrP9MDH5MBPbIqV92AaeXatLxBI9gBaebbnrfifHhDYfgasaacH8akY=wiFfYdH8Gipec8Eeeu0xXdbba9frFj0=OqFfea0dXdd9vqai=hGuQ8kuc9pgc9s8qqaq=dirpe0xb9q8qiLsFr0=vr0=vr0dc8meaabaqaciaacaGaaeqabaqabeGadaaakeaadaWcaaqaaiabigdaXaqaaiabikdaYaaadaqbdaqaaiabdEha3bGaayzcSlaawQa7amaaCaaaleqabaGaeGOmaidaaaaa@345B@

Constrained to {yi−(wtxi)−b≤ε(wtxi)+b−yi≤ε     (16)
 MathType@MTEF@5@5@+=feaafiart1ev1aaatCvAUfKttLearuWrP9MDH5MBPbIqV92AaeXatLxBI9gBaebbnrfifHhDYfgasaacH8akY=wiFfYdH8Gipec8Eeeu0xXdbba9frFj0=OqFfea0dXdd9vqai=hGuQ8kuc9pgc9s8qqaq=dirpe0xb9q8qiLsFr0=vr0=vr0dc8meaabaqaciaacaGaaeqabaqabeGadaaakeaacqqGdbWqcqqGVbWBcqqGUbGBcqqGZbWCcqqG0baDcqqGYbGCcqqGHbqycqqGPbqAcqqGUbGBcqqGLbqzcqqGKbazcqqGGaaicqqG0baDcqqGVbWBcqqGGaaidaGabeqaauaabaqaceaaaeaacqWG5bqEdaWgaaWcbaGaemyAaKgabeaakiabgkHiTmaabmaabaGaem4DaC3aaWbaaSqabeaacqWG0baDaaGccqWG4baEdaWgaaWcbaGaemyAaKgabeaaaOGaayjkaiaawMcaaiabgkHiTiabdkgaIjabgsMiJIGaciab=v7aLbqaamaabmaabaGaem4DaC3aaWbaaSqabeaacqWG0baDaaGccqWG4baEdaWgaaWcbaGaemyAaKgabeaaaOGaayjkaiaawMcaaiabgUcaRiabdkgaIjabgkHiTiabdMha5naaBaaaleaacqWGPbqAaeqaaOGaeyizImQae8xTdugaaiaaxMaacaWLjaGaeiikaGIaeGymaeJaeGOnayJaeiykaKcacaGL7baaaaa@684F@

In (16) there is a function *f *which, with *ε *precision, approximates all pairs (*x*_i_, *y*_*i*_). But there are cases where it is necessary to allow for some errors. To solve this problem, one can introduce slack variables *ξ*_*i*_, ξi∗
 MathType@MTEF@5@5@+=feaafiart1ev1aaatCvAUfKttLearuWrP9MDH5MBPbIqV92AaeXatLxBI9gBaebbnrfifHhDYfgasaacH8akY=wiFfYdH8Gipec8Eeeu0xXdbba9frFj0=OqFfea0dXdd9vqai=hGuQ8kuc9pgc9s8qqaq=dirpe0xb9q8qiLsFr0=vr0=vr0dc8meaabaqaciaacaGaaeqabaqabeGadaaakeaacqaH+oaEdaqhaaWcbaGaemyAaKgabaGaey4fIOcaaaaa@30E6@ to deal with unfeasible constraints of the optimization problem (16) arriving at the formulation stated in [41]

Minimize 12‖w‖2
 MathType@MTEF@5@5@+=feaafiart1ev1aaatCvAUfKttLearuWrP9MDH5MBPbIqV92AaeXatLxBI9gBaebbnrfifHhDYfgasaacH8akY=wiFfYdH8Gipec8Eeeu0xXdbba9frFj0=OqFfea0dXdd9vqai=hGuQ8kuc9pgc9s8qqaq=dirpe0xb9q8qiLsFr0=vr0=vr0dc8meaabaqaciaacaGaaeqabaqabeGadaaakeaadaWcaaqaaiabigdaXaqaaiabikdaYaaadaqbdaqaaiabdEha3bGaayzcSlaawQa7amaaCaaaleqabaGaeGOmaidaaaaa@345B@ + *C *∑(*ξ*_*i *_+ ξi∗
 MathType@MTEF@5@5@+=feaafiart1ev1aaatCvAUfKttLearuWrP9MDH5MBPbIqV92AaeXatLxBI9gBaebbnrfifHhDYfgasaacH8akY=wiFfYdH8Gipec8Eeeu0xXdbba9frFj0=OqFfea0dXdd9vqai=hGuQ8kuc9pgc9s8qqaq=dirpe0xb9q8qiLsFr0=vr0=vr0dc8meaabaqaciaacaGaaeqabaqabeGadaaakeaacqaH+oaEdaqhaaWcbaGaemyAaKgabaGaey4fIOcaaaaa@30E6@)

Constrained to {yi−(wtxi)−b≤ε+ξi(wtxi)+b−yi≤ε+ξi*ξi,ξi*≥0     (17)
 MathType@MTEF@5@5@+=feaafiart1ev1aaatCvAUfKttLearuWrP9MDH5MBPbIqV92AaeXatLxBI9gBaebbnrfifHhDYfgasaacH8akY=wiFfYdH8Gipec8Eeeu0xXdbba9frFj0=OqFfea0dXdd9vqai=hGuQ8kuc9pgc9s8qqaq=dirpe0xb9q8qiLsFr0=vr0=vr0dc8meaabaqaciaacaGaaeqabaqabeGadaaakeaacqqGdbWqcqqGVbWBcqqGUbGBcqqGZbWCcqqG0baDcqqGYbGCcqqGHbqycqqGPbqAcqqGUbGBcqqGLbqzcqqGKbazcqqGGaaicqqG0baDcqqGVbWBcqqGGaaidaGabeqaauaabaqadeaaaeaacqWG5bqEdaWgaaWcbaGaemyAaKgabeaakiabgkHiTmaabmaabaGaem4DaC3aaWbaaSqabeaacqWG0baDaaGccqWG4baEdaWgaaWcbaGaemyAaKgabeaaaOGaayjkaiaawMcaaiabgkHiTiabdkgaIjabgsMiJIGaciab=v7aLjabgUcaRiab=57a4naaBaaaleaacqWGPbqAaeqaaaGcbaWaaeWaaeaacqWG3bWDdaahaaWcbeqaaiabdsha0baakiabdIha4naaBaaaleaacqWGPbqAaeqaaaGccaGLOaGaayzkaaGaey4kaSIaemOyaiMaeyOeI0IaemyEaK3aaSbaaSqaaiabdMgaPbqabaGccqGHKjYOcqWF1oqzcqGHRaWkcqWF+oaEdaqhaaWcbaGaemyAaKgabaGaeiOkaOcaaaGcbaGae8NVdG3aaSbaaSqaaiabdMgaPbqabaGccqGGSaalcqWF+oaEdaqhaaWcbaGaemyAaKgabaGaeiOkaOcaaOGaeyyzImRaeGimaadaaaGaay5EaaGaaCzcaiaaxMaacqGGOaakcqaIXaqmcqaI3aWncqGGPaqkaaa@7CA1@

where the constant *C *> 0 is the trade-off between the amount up to which deviations larger than *ε *are tolerated, maintaining the flatness of *f*. This corresponds to dealing with the *ε*-insensitive loss function |*ξ*|_*ε *_:

|ξ|ε:={0 if|ξ|≤ε|ξ|−ε otherwise     (18)
 MathType@MTEF@5@5@+=feaafiart1ev1aaatCvAUfKttLearuWrP9MDH5MBPbIqV92AaeXatLxBI9gBaebbnrfifHhDYfgasaacH8akY=wiFfYdH8Gipec8Eeeu0xXdbba9frFj0=OqFfea0dXdd9vqai=hGuQ8kuc9pgc9s8qqaq=dirpe0xb9q8qiLsFr0=vr0=vr0dc8meaabaqaciaacaGaaeqabaqabeGadaaakeaadaabdaqaaGGaciab=57a4bGaay5bSlaawIa7amaaBaaaleaacqWF1oqzaeqaaOGaeiOoaOJaeyypa0ZaaiqabeaafaqaaeGabaaabaGaeGimaaJaeeiiaaIaemyAaKMaemOzay2aaqWaaeaacqWF+oaEaiaawEa7caGLiWoacqGHKjYOcqWF1oqzaeaadaabdaqaaiab=57a4bGaay5bSlaawIa7aiabgkHiTiab=v7aLjabbccaGiabd+gaVjabdsha0jabdIgaOjabdwgaLjabdkhaYjabdEha3jabdMgaPjabdohaZjabdwgaLbaaaiaawUhaaiaaxMaacaWLjaGaeiikaGIaeGymaeJaeGioaGJaeiykaKcaaa@5CD4@

It is necessary to construct a Lagrange function from the primal objective function and the corresponding constraints by introducing a dual set of variables. According to Mangasarian (1969) [42], McCormick (1983) [43], and Vanderbei (1997) [44] it follows that:

L:=12‖w‖2+C∑i=1l(ξi+ξi*)−∑i=1l(ηiξi+ηi*ξi*)−∑i=1lαi(ε+ξi−yi+(wtxi)+b)−∑αi*(ε+ξi*+yi−(wtxi)−b)     (19)
 MathType@MTEF@5@5@+=feaafiart1ev1aaatCvAUfKttLearuWrP9MDH5MBPbIqV92AaeXatLxBI9gBaebbnrfifHhDYfgasaacH8akY=wiFfYdH8Gipec8Eeeu0xXdbba9frFj0=OqFfea0dXdd9vqai=hGuQ8kuc9pgc9s8qqaq=dirpe0xb9q8qiLsFr0=vr0=vr0dc8meaabaqaciaacaGaaeqabaqabeGadaaakeaacqWGmbatcqGG6aGocqGH9aqpdaWcaaqaaiabigdaXaqaaiabikdaYaaadaqbdaqaaiabdEha3bGaayzcSlaawQa7amaaCaaaleqabaGaeGOmaidaaOGaey4kaSIaem4qam0aaabCaeaadaqadaqaaGGaciab=57a4naaBaaaleaacqWGPbqAaeqaaOGaey4kaSIae8NVdG3aa0baaSqaaiabdMgaPbqaaiabcQcaQaaaaOGaayjkaiaawMcaaiabgkHiTaWcbaGaemyAaKMaeyypa0JaeGymaedabaGaemiBaWganiabggHiLdGcdaaeWbqaamaabmaabaGae83TdG2aaSbaaSqaaiabdMgaPbqabaGccqWF+oaEdaWgaaWcbaGaemyAaKgabeaakiabgUcaRiab=D7aOnaaDaaaleaacqWGPbqAaeaacqGGQaGkaaGccqWF+oaEdaqhaaWcbaGaemyAaKgabaGaeiOkaOcaaaGccaGLOaGaayzkaaaaleaacqWGPbqAcqGH9aqpcqaIXaqmaeaacqWGSbaBa0GaeyyeIuoakiabgkHiTmaaqahabaGae8xSde2aaSbaaSqaaiabdMgaPbqabaaabaGaemyAaKMaeyypa0JaeGymaedabaGaemiBaWganiabggHiLdGcdaqadaqaaiab=v7aLjabgUcaRiab=57a4naaBaaaleaacqWGPbqAaeqaaOGaeyOeI0IaemyEaK3aaSbaaSqaaiabdMgaPbqabaGccqGHRaWkdaqadaqaaiabdEha3naaCaaaleqabaGaemiDaqhaaOGaemiEaG3aaSbaaSqaaiabdMgaPbqabaaakiaawIcacaGLPaaacqGHRaWkcqWGIbGyaiaawIcacaGLPaaacqGHsisldaaeabqaaiab=f7aHnaaDaaaleaacqWGPbqAaeaacqGGQaGkaaaabeqab0GaeyyeIuoakmaabmaabaGae8xTduMaey4kaSIae8NVdG3aa0baaSqaaiabdMgaPbqaaiabcQcaQaaakiabgUcaRiabdMha5naaBaaaleaacqWGPbqAaeqaaOGaeyOeI0YaaeWaaeaacqWG3bWDdaahaaWcbeqaaiabdsha0baakiabdIha4naaBaaaleaacqWGPbqAaeqaaaGccaGLOaGaayzkaaGaeyOeI0IaemOyaigacaGLOaGaayzkaaGaaCzcaiaaxMaacqGGOaakcqaIXaqmcqaI5aqocqGGPaqkaaa@A784@

where *L *is the Lagrangian and *η*_*i*_, ηi*
 MathType@MTEF@5@5@+=feaafiart1ev1aaatCvAUfKttLearuWrP9MDH5MBPbIqV92AaeXatLxBI9gBaebbnrfifHhDYfgasaacH8akY=wiFfYdH8Gipec8Eeeu0xXdbba9frFj0=OqFfea0dXdd9vqai=hGuQ8kuc9pgc9s8qqaq=dirpe0xb9q8qiLsFr0=vr0=vr0dc8meaabaqaciaacaGaaeqabaqabeGadaaakeaaiiGacqWF3oaAdaqhaaWcbaGaemyAaKgabaGaeiOkaOcaaaaa@30C3@, *α*_*i*_, αi*
 MathType@MTEF@5@5@+=feaafiart1ev1aaatCvAUfKttLearuWrP9MDH5MBPbIqV92AaeXatLxBI9gBaebbnrfifHhDYfgasaacH8akY=wiFfYdH8Gipec8Eeeu0xXdbba9frFj0=OqFfea0dXdd9vqai=hGuQ8kuc9pgc9s8qqaq=dirpe0xb9q8qiLsFr0=vr0=vr0dc8meaabaqaciaacaGaaeqabaqabeGadaaakeaaiiGacqWFXoqydaqhaaWcbaGaemyAaKgabaGaeiOkaOcaaaaa@30B6@ are Lagrange multipliers. Hence the dual variables in (19) have to satisfy

αi(*),ηi(*)≥0     (20)
 MathType@MTEF@5@5@+=feaafiart1ev1aaatCvAUfKttLearuWrP9MDH5MBPbIqV92AaeXatLxBI9gBaebbnrfifHhDYfgasaacH8akY=wiFfYdH8Gipec8Eeeu0xXdbba9frFj0=OqFfea0dXdd9vqai=hGuQ8kuc9pgc9s8qqaq=dirpe0xb9q8qiLsFr0=vr0=vr0dc8meaabaqaciaacaGaaeqabaqabeGadaaakeaaiiGacqWFXoqydaqhaaWcbaGaemyAaKgabaGaeiikaGIaeiOkaOIaeiykaKcaaOGaeiilaWIae83TdG2aa0baaSqaaiabdMgaPbqaaiabcIcaOiabcQcaQiabcMcaPaaakiabgwMiZkabicdaWiaaxMaacaWLjaGaeiikaGIaeGOmaiJaeGimaaJaeiykaKcaaa@40A3@

Note that we refer to *α*_*i *_and αi*
 MathType@MTEF@5@5@+=feaafiart1ev1aaatCvAUfKttLearuWrP9MDH5MBPbIqV92AaeXatLxBI9gBaebbnrfifHhDYfgasaacH8akY=wiFfYdH8Gipec8Eeeu0xXdbba9frFj0=OqFfea0dXdd9vqai=hGuQ8kuc9pgc9s8qqaq=dirpe0xb9q8qiLsFr0=vr0=vr0dc8meaabaqaciaacaGaaeqabaqabeGadaaakeaaiiGacqWFXoqydaqhaaWcbaGaemyAaKgabaGaeiOkaOcaaaaa@30B6@ as αi(∗)
 MathType@MTEF@5@5@+=feaafiart1ev1aaatCvAUfKttLearuWrP9MDH5MBPbIqV92AaeXatLxBI9gBaebbnrfifHhDYfgasaacH8akY=wiFfYdH8Gipec8Eeeu0xXdbba9frFj0=OqFfea0dXdd9vqai=hGuQ8kuc9pgc9s8qqaq=dirpe0xb9q8qiLsFr0=vr0=vr0dc8meaabaqaciaacaGaaeqabaqabeGadaaakeaaiiGacqWFXoqydaqhaaWcbaGaemyAaKgabaGaeiikaGIaey4fIOIaeiykaKcaaaaa@327B@.

From the saddle point condition, the partial derivatives of *L *related to (*w*, *b*, *ξ*_*i*_, ξi∗
 MathType@MTEF@5@5@+=feaafiart1ev1aaatCvAUfKttLearuWrP9MDH5MBPbIqV92AaeXatLxBI9gBaebbnrfifHhDYfgasaacH8akY=wiFfYdH8Gipec8Eeeu0xXdbba9frFj0=OqFfea0dXdd9vqai=hGuQ8kuc9pgc9s8qqaq=dirpe0xb9q8qiLsFr0=vr0=vr0dc8meaabaqaciaacaGaaeqabaqabeGadaaakeaacqaH+oaEdaqhaaWcbaGaemyAaKgabaGaey4fIOcaaaaa@30E6@) have to vanish for optimality.

∂bL=∑i=1l(αi*−αi)=0     (21)
 MathType@MTEF@5@5@+=feaafiart1ev1aaatCvAUfKttLearuWrP9MDH5MBPbIqV92AaeXatLxBI9gBaebbnrfifHhDYfgasaacH8akY=wiFfYdH8Gipec8Eeeu0xXdbba9frFj0=OqFfea0dXdd9vqai=hGuQ8kuc9pgc9s8qqaq=dirpe0xb9q8qiLsFr0=vr0=vr0dc8meaabaqaciaacaGaaeqabaqabeGadaaakeaacqGHciITdaWgaaWcbaGaemOyaigabeaakiabdYeamjabg2da9maaqahabaWaaeWaaeaaiiGacqWFXoqydaqhaaWcbaGaemyAaKgabaGaeiOkaOcaaOGaeyOeI0Iae8xSde2aaSbaaSqaaiabdMgaPbqabaaakiaawIcacaGLPaaacqGH9aqpcqaIWaamcaWLjaGaaCzcaiabcIcaOiabikdaYiabigdaXiabcMcaPaWcbaGaemyAaKMaeyypa0JaeGymaedabaGaemiBaWganiabggHiLdaaaa@4931@

∂wL=w−∑i=1l(αi−αi*)xi=0     (22)
 MathType@MTEF@5@5@+=feaafiart1ev1aaatCvAUfKttLearuWrP9MDH5MBPbIqV92AaeXatLxBI9gBaebbnrfifHhDYfgasaacH8akY=wiFfYdH8Gipec8Eeeu0xXdbba9frFj0=OqFfea0dXdd9vqai=hGuQ8kuc9pgc9s8qqaq=dirpe0xb9q8qiLsFr0=vr0=vr0dc8meaabaqaciaacaGaaeqabaqabeGadaaakeaacqGHciITdaWgaaWcbaGaem4DaChabeaakiabdYeamjabg2da9iabdEha3jabgkHiTmaaqahabaWaaeWaaeaaiiGacqWFXoqydaWgaaWcbaGaemyAaKgabeaakiabgkHiTiab=f7aHnaaDaaaleaacqWGPbqAaeaacqGGQaGkaaaakiaawIcacaGLPaaaaSqaaiabdMgaPjabg2da9iabigdaXaqaaiabdYgaSbqdcqGHris5aOGaemiEaG3aaSbaaSqaaiabdMgaPbqabaGccqGH9aqpcqaIWaamcaWLjaGaaCzcaiabcIcaOiabikdaYiabikdaYiabcMcaPaaa@4ED5@

∂ξi(*)L=C−αi(*)−ηi(*)=0     (23)
 MathType@MTEF@5@5@+=feaafiart1ev1aaatCvAUfKttLearuWrP9MDH5MBPbIqV92AaeXatLxBI9gBaebbnrfifHhDYfgasaacH8akY=wiFfYdH8Gipec8Eeeu0xXdbba9frFj0=OqFfea0dXdd9vqai=hGuQ8kuc9pgc9s8qqaq=dirpe0xb9q8qiLsFr0=vr0=vr0dc8meaabaqaciaacaGaaeqabaqabeGadaaakeaacqGHciITdaWgaaWcbaacciGae8NVdG3aaSbaaWqaaiabdMgaPbqabaaaleqaaOGaeiikaGIaeiOkaOIaeiykaKIaemitaWKaeyypa0Jaem4qamKaeyOeI0Iae8xSde2aa0baaSqaaiabdMgaPbqaaiabcIcaOiabcQcaQiabcMcaPaaakiabgkHiTiab=D7aOnaaDaaaleaacqWGPbqAaeaacqGGOaakcqGGQaGkcqGGPaqkaaGccqGH9aqpcqaIWaamcaWLjaGaaCzcaiabcIcaOiabikdaYiabiodaZiabcMcaPaaa@4B94@

From the substitution of (21), (22) and (23) into (19) we obtain a dual optimization problem.

Maximize {−12∑i,j=1l(αi−αi*)(αj−αj*)(xitxj)−ε∑i=1l(αi−αi*)+∑i=1lyi(αi−αi*)     (24)
 MathType@MTEF@5@5@+=feaafiart1ev1aaatCvAUfKttLearuWrP9MDH5MBPbIqV92AaeXatLxBI9gBaebbnrfifHhDYfgasaacH8akY=wiFfYdH8Gipec8Eeeu0xXdbba9frFj0=OqFfea0dXdd9vqai=hGuQ8kuc9pgc9s8qqaq=dirpe0xb9q8qiLsFr0=vr0=vr0dc8meaabaqaciaacaGaaeqabaqabeGadaaakeaacqqGnbqtcqqGHbqycqqG4baEcqqGPbqAcqqGTbqBcqqGPbqAcqqG6bGEcqqGLbqzcqqGGaaidaGabeqaauaabaqaceaaaeaacqGHsisldaWcaaqaaiabigdaXaqaaiabikdaYaaadaaeWbqaamaabmaabaacciGae8xSde2aaSbaaSqaaiabdMgaPbqabaGccqGHsislcqWFXoqydaqhaaWcbaGaemyAaKgabaGaeiOkaOcaaaGccaGLOaGaayzkaaaaleaacqWGPbqAcqGGSaalcqWGQbGAcqGH9aqpcqaIXaqmaeaacqWGSbaBa0GaeyyeIuoakmaabmaabaGae8xSde2aaSbaaSqaaiabdQgaQbqabaGccqGHsislcqWFXoqydaqhaaWcbaGaemOAaOgabaGaeiOkaOcaaaGccaGLOaGaayzkaaWaaeWaaeaacqWG4baEdaqhaaWcbaGaemyAaKgabaGaemiDaqhaaOGaemiEaG3aaSbaaSqaaiabdQgaQbqabaaakiaawIcacaGLPaaaaeaacqGHsislcqWF1oqzdaaeWbqaamaabmaabaGae8xSde2aaSbaaSqaaiabdMgaPbqabaGccqGHsislcqWFXoqydaqhaaWcbaGaemyAaKgabaGaeiOkaOcaaaGccaGLOaGaayzkaaaaleaacqWGPbqAcqGH9aqpcqaIXaqmaeaacqWGSbaBa0GaeyyeIuoakiabgUcaRmaaqahabaGaemyEaK3aaSbaaSqaaiabdMgaPbqabaaabaGaemyAaKMaeyypa0JaeGymaedabaGaemiBaWganiabggHiLdGcdaqadaqaaiab=f7aHnaaBaaaleaacqWGPbqAaeqaaOGaeyOeI0Iae8xSde2aa0baaSqaaiabdMgaPbqaaiabcQcaQaaaaOGaayjkaiaawMcaaaaaaiaawUhaaiaaxMaacaWLjaGaeiikaGIaeGOmaiJaeGinaqJaeiykaKcaaa@8E7B@

Subject to ∑i=1l(αi−αi*)=0
 MathType@MTEF@5@5@+=feaafiart1ev1aaatCvAUfKttLearuWrP9MDH5MBPbIqV92AaeXatLxBI9gBaebbnrfifHhDYfgasaacH8akY=wiFfYdH8Gipec8Eeeu0xXdbba9frFj0=OqFfea0dXdd9vqai=hGuQ8kuc9pgc9s8qqaq=dirpe0xb9q8qiLsFr0=vr0=vr0dc8meaabaqaciaacaGaaeqabaqabeGadaaakeaadaaeWbqaamaabmaabaacciGae8xSde2aaSbaaSqaaiabdMgaPbqabaGccqGHsislcqWFXoqydaqhaaWcbaGaemyAaKgabaGaeiOkaOcaaaGccaGLOaGaayzkaaGaeyypa0JaeGimaadaleaacqWGPbqAcqGH9aqpcqaIXaqmaeaacqWGSbaBa0GaeyyeIuoaaaa@3F49@ and *α*_*i*_, αi*
 MathType@MTEF@5@5@+=feaafiart1ev1aaatCvAUfKttLearuWrP9MDH5MBPbIqV92AaeXatLxBI9gBaebbnrfifHhDYfgasaacH8akY=wiFfYdH8Gipec8Eeeu0xXdbba9frFj0=OqFfea0dXdd9vqai=hGuQ8kuc9pgc9s8qqaq=dirpe0xb9q8qiLsFr0=vr0=vr0dc8meaabaqaciaacaGaaeqabaqabeGadaaakeaaiiGacqWFXoqydaqhaaWcbaGaemyAaKgabaGaeiOkaOcaaaaa@30B6@ ∈ [0, *C*]

Equation (22) can be rewritten as follows

w=∑i=1l(αi−αi*)xi
 MathType@MTEF@5@5@+=feaafiart1ev1aaatCvAUfKttLearuWrP9MDH5MBPbIqV92AaeXatLxBI9gBaebbnrfifHhDYfgasaacH8akY=wiFfYdH8Gipec8Eeeu0xXdbba9frFj0=OqFfea0dXdd9vqai=hGuQ8kuc9pgc9s8qqaq=dirpe0xb9q8qiLsFr0=vr0=vr0dc8meaabaqaciaacaGaaeqabaqabeGadaaakeaacqWG3bWDcqGH9aqpdaaeWbqaamaabmaabaacciGae8xSde2aaSbaaSqaaiabdMgaPbqabaGccqGHsislcqWFXoqydaqhaaWcbaGaemyAaKgabaGaeiOkaOcaaaGccaGLOaGaayzkaaaaleaacqWGPbqAcqGH9aqpcqaIXaqmaeaacqWGSbaBa0GaeyyeIuoakiabdIha4naaBaaaleaacqWGPbqAaeqaaaaa@42DC@, thus f(x)=∑i=1l(αi−αi*)(xtx)+b
 MathType@MTEF@5@5@+=feaafiart1ev1aaatCvAUfKttLearuWrP9MDH5MBPbIqV92AaeXatLxBI9gBaebbnrfifHhDYfgasaacH8akY=wiFfYdH8Gipec8Eeeu0xXdbba9frFj0=OqFfea0dXdd9vqai=hGuQ8kuc9pgc9s8qqaq=dirpe0xb9q8qiLsFr0=vr0=vr0dc8meaabaqaciaacaGaaeqabaqabeGadaaakeaacqWGMbGzcqGGOaakcqWG4baEcqGGPaqkcqGH9aqpdaaeWbqaamaabmaabaacciGae8xSde2aaSbaaSqaaiabdMgaPbqabaGccqGHsislcqWFXoqydaqhaaWcbaGaemyAaKgabaGaeiOkaOcaaaGccaGLOaGaayzkaaWaaeWaaeaacqWG4baEdaahaaWcbeqaaiabdsha0baakiabdIha4bGaayjkaiaawMcaaaWcbaGaemyAaKMaeyypa0JaeGymaedabaGaemiBaWganiabggHiLdGccqGHRaWkcqWGIbGyaaa@4B37@     (25)

This is the Support Vector expansion, i.e., the description of *w *as a linear combination of *x*_*i*_.

To compute *b*, it is necessary to use Karush-Kuhn-Tucker (KKT) conditions [45,46]. These authors state that at the point of the solution the product between dual variables and constraints has to vanish.

*α*_*i *_(*ε *+ *ξ*_*i *_- *y*_*i *_+ (*w*^*t *^*x*_*i*_) + *b*) = 0     (26)

αi*
 MathType@MTEF@5@5@+=feaafiart1ev1aaatCvAUfKttLearuWrP9MDH5MBPbIqV92AaeXatLxBI9gBaebbnrfifHhDYfgasaacH8akY=wiFfYdH8Gipec8Eeeu0xXdbba9frFj0=OqFfea0dXdd9vqai=hGuQ8kuc9pgc9s8qqaq=dirpe0xb9q8qiLsFr0=vr0=vr0dc8meaabaqaciaacaGaaeqabaqabeGadaaakeaaiiGacqWFXoqydaqhaaWcbaGaemyAaKgabaGaeiOkaOcaaaaa@30B6@ (*ε *+ ξi∗
 MathType@MTEF@5@5@+=feaafiart1ev1aaatCvAUfKttLearuWrP9MDH5MBPbIqV92AaeXatLxBI9gBaebbnrfifHhDYfgasaacH8akY=wiFfYdH8Gipec8Eeeu0xXdbba9frFj0=OqFfea0dXdd9vqai=hGuQ8kuc9pgc9s8qqaq=dirpe0xb9q8qiLsFr0=vr0=vr0dc8meaabaqaciaacaGaaeqabaqabeGadaaakeaacqaH+oaEdaqhaaWcbaGaemyAaKgabaGaey4fIOcaaaaa@30E6@ + *y*_*i *_- (*w*^*t *^*x*_*i*_) - *b*) = 0

and

(*C *- *α*_*i*_)*ξ*_*i *_= 0     (27)

(*C *- αi*
 MathType@MTEF@5@5@+=feaafiart1ev1aaatCvAUfKttLearuWrP9MDH5MBPbIqV92AaeXatLxBI9gBaebbnrfifHhDYfgasaacH8akY=wiFfYdH8Gipec8Eeeu0xXdbba9frFj0=OqFfea0dXdd9vqai=hGuQ8kuc9pgc9s8qqaq=dirpe0xb9q8qiLsFr0=vr0=vr0dc8meaabaqaciaacaGaaeqabaqabeGadaaakeaaiiGacqWFXoqydaqhaaWcbaGaemyAaKgabaGaeiOkaOcaaaaa@30B6@) ξi∗
 MathType@MTEF@5@5@+=feaafiart1ev1aaatCvAUfKttLearuWrP9MDH5MBPbIqV92AaeXatLxBI9gBaebbnrfifHhDYfgasaacH8akY=wiFfYdH8Gipec8Eeeu0xXdbba9frFj0=OqFfea0dXdd9vqai=hGuQ8kuc9pgc9s8qqaq=dirpe0xb9q8qiLsFr0=vr0=vr0dc8meaabaqaciaacaGaaeqabaqabeGadaaakeaacqaH+oaEdaqhaaWcbaGaemyAaKgabaGaey4fIOcaaaaa@30E6@ = 0

From (26) and (27) it follows that:

(i) Only samples (*x*_*i*_, *y*_*i*_) with corresponding ∂bL=∑i=1l(αi*−αi)=0     (21)
 MathType@MTEF@5@5@+=feaafiart1ev1aaatCvAUfKttLearuWrP9MDH5MBPbIqV92AaeXatLxBI9gBaebbnrfifHhDYfgasaacH8akY=wiFfYdH8Gipec8Eeeu0xXdbba9frFj0=OqFfea0dXdd9vqai=hGuQ8kuc9pgc9s8qqaq=dirpe0xb9q8qiLsFr0=vr0=vr0dc8meaabaqaciaacaGaaeqabaqabeGadaaakeaacqGHciITdaWgaaWcbaGaemOyaigabeaakiabdYeamjabg2da9maaqahabaWaaeWaaeaaiiGacqWFXoqydaqhaaWcbaGaemyAaKgabaGaeiOkaOcaaOGaeyOeI0Iae8xSde2aaSbaaSqaaiabdMgaPbqabaaakiaawIcacaGLPaaacqGH9aqpcqaIWaamcaWLjaGaaCzcaiabcIcaOiabikdaYiabigdaXiabcMcaPaWcbaGaemyAaKMaeyypa0JaeGymaedabaGaemiBaWganiabggHiLdaaaa@4931@ = *C *lie outside the *ε*-insensitive tube;

(ii) *α*_*i *_αi*
 MathType@MTEF@5@5@+=feaafiart1ev1aaatCvAUfKttLearuWrP9MDH5MBPbIqV92AaeXatLxBI9gBaebbnrfifHhDYfgasaacH8akY=wiFfYdH8Gipec8Eeeu0xXdbba9frFj0=OqFfea0dXdd9vqai=hGuQ8kuc9pgc9s8qqaq=dirpe0xb9q8qiLsFr0=vr0=vr0dc8meaabaqaciaacaGaaeqabaqabeGadaaakeaaiiGacqWFXoqydaqhaaWcbaGaemyAaKgabaGaeiOkaOcaaaaa@30B6@ = 0

From (i) and (ii), it is possible to conclude that

*ε *- *y*_*i *_+ (*w*^*t *^*x*_*i*_) + *b *≥ 0 and *ξ*_*i *_= 0 if *α*_*i *_<*C *    (28)

*ε *- *y*_*i *_+ (*w*^*t *^*x*_*i*_) + *b *≤ 0     if *α*_*i *_> 0     (29)

In conjunction with an analogous analysis on αi*
 MathType@MTEF@5@5@+=feaafiart1ev1aaatCvAUfKttLearuWrP9MDH5MBPbIqV92AaeXatLxBI9gBaebbnrfifHhDYfgasaacH8akY=wiFfYdH8Gipec8Eeeu0xXdbba9frFj0=OqFfea0dXdd9vqai=hGuQ8kuc9pgc9s8qqaq=dirpe0xb9q8qiLsFr0=vr0=vr0dc8meaabaqaciaacaGaaeqabaqabeGadaaakeaaiiGacqWFXoqydaqhaaWcbaGaemyAaKgabaGaeiOkaOcaaaaa@30B6@

max{-*ε *+ *y*_*i *_- (*w*^*t *^*x*_*i*_)|*α*_*i *_<*C or *αi*
 MathType@MTEF@5@5@+=feaafiart1ev1aaatCvAUfKttLearuWrP9MDH5MBPbIqV92AaeXatLxBI9gBaebbnrfifHhDYfgasaacH8akY=wiFfYdH8Gipec8Eeeu0xXdbba9frFj0=OqFfea0dXdd9vqai=hGuQ8kuc9pgc9s8qqaq=dirpe0xb9q8qiLsFr0=vr0=vr0dc8meaabaqaciaacaGaaeqabaqabeGadaaakeaaiiGacqWFXoqydaqhaaWcbaGaemyAaKgabaGaeiOkaOcaaaaa@30B6@ > 0} ≤ *b *≤ min{-*ε *+ *y*_*i *_- (*w*^*t *^*x*_*i*_)|*α*_*i *_> 0 *or *αi*
 MathType@MTEF@5@5@+=feaafiart1ev1aaatCvAUfKttLearuWrP9MDH5MBPbIqV92AaeXatLxBI9gBaebbnrfifHhDYfgasaacH8akY=wiFfYdH8Gipec8Eeeu0xXdbba9frFj0=OqFfea0dXdd9vqai=hGuQ8kuc9pgc9s8qqaq=dirpe0xb9q8qiLsFr0=vr0=vr0dc8meaabaqaciaacaGaaeqabaqabeGadaaakeaaiiGacqWFXoqydaqhaaWcbaGaemyAaKgabaGaeiOkaOcaaaaa@30B6@ <*C*}     (30)

If some αi*
 MathType@MTEF@5@5@+=feaafiart1ev1aaatCvAUfKttLearuWrP9MDH5MBPbIqV92AaeXatLxBI9gBaebbnrfifHhDYfgasaacH8akY=wiFfYdH8Gipec8Eeeu0xXdbba9frFj0=OqFfea0dXdd9vqai=hGuQ8kuc9pgc9s8qqaq=dirpe0xb9q8qiLsFr0=vr0=vr0dc8meaabaqaciaacaGaaeqabaqabeGadaaakeaaiiGacqWFXoqydaqhaaWcbaGaemyAaKgabaGaeiOkaOcaaaaa@30B6@ ∈ (0, *C*) the inequalities become equalities.

To point out the sparsity of the SV expansion: from (26), the Lagrange multipliers may be nonzero only for |*f *(*x*_*i*_) - *y*_*i*_| ≥ *ε*.

Therefore, we have a sparse expansion of *w *in terms of *x*_*i *_[47].

## Abbreviations

LR: Loess Regression

SS: Splines Smoothing

WS: Wavelets Smoothing

KR: Kernel Regression

SVR: Support Vector Regression

## Authors' contributions

**AF **– has made substantial contributions to conception and design of the study, analysis and interpretation of data and has been involved in drafting of the manuscript.

**JRS **– has made substantial contributions to conception and design of the study, analysis and interpretation of data and has been involved in drafting of the manuscript.

**LOR **– acquisition of the benchmark data and has been involved in drafting parts of the manuscript.

**CEF **– has discussed the results and critically revised the manuscript for important intellectual content and has given the final approval of the version to be published.

**MCS **– has directed the work on differentially expressed genes using the CodeLink™ platform and critically revised the manuscript for important intellectual content and has given the final approval of the version to be published.
